# Vacuum‐Assisted Delivery and Neonatal Intensive Care Unit Admission: A Retrospective Case‐Control Study at a Lebanese Hospital

**DOI:** 10.1155/ijpe/7350932

**Published:** 2026-09-24

**Authors:** Maribelle Azar, Tara Chahwan, Souheil Hallit, Habib Barakat

**Affiliations:** ^1^ School of Medicine and Medical Sciences, Holy Spirit University of Kaslik, Jounieh, Lebanon, usek.edu.lb; ^2^ Applied Science Research Center, Applied Science Private University, Amman, Jordan, asu.edu.jo; ^3^ Obstetrics and Gynecology Department, Notre Dame des Secours University Hospital, Byblos, Lebanon

**Keywords:** Lebanon, maternal–fetal factors, neonatal outcomes, NICU admission, retrospective case-control study, vacuum-assisted vaginal delivery (VAVD)

## Abstract

**Background:**

Vacuum‐assisted vaginal delivery (VAVD) is a common obstetric intervention used to facilitate childbirth. In Lebanon, the practice remains inconsistently applied. Standardized national guidelines and their impact on neonatal outcomes, particularly neonatal intensive care unit (NICU) admissions, remain insufficiently explored.

**Objective:**

To examine the association between VAVD and NICU admission in a tertiary hospital in Lebanon.

**Methods:**

A retrospective hospital‐based case‐control study was conducted between January 2021 and December 2023. Cases were defined as neonates admitted to the NICU after delivery, and controls as neonates not requiring NICU admission. Prior exposure (mode of delivery: VAVD vs. spontaneous vaginal delivery) and maternal, obstetric, and neonatal variables were compared between the two groups. All live‐born neonates (*n* = 488), including both term and preterm infants, were screened. Data were extracted from medical records, and multivariate logistic regression was used to assess the association between VAVD and NICU admission.

**Results:**

VAVD was not significantly associated with NICU admission (adjusted OR = 2.11; 95% CI: 1.00–4.49; *p* = 0.051).

**Conclusion:**

In this setting, VAVD was not associated with an increased likelihood of NICU admission. Strengthening guidelines and training for VAVD use may help optimize neonatal outcomes in Lebanon.

## 1. Introduction

Neonatal intensive care unit (NICU) admission is widely represented as a key indicator of neonatal morbidity. Access to NICU services improves outcomes for high‐risk infants, particularly those born preterm or with significant medical complications [1]. Multiple maternal, obstetric, and neonatal factors have been associated with NICU admission. Delivery‐related complications such as shoulder dystocia and mode of delivery are important contributors, whereas neonatal characteristics, particularly gestational age, prematurity, and sex also influence the likelihood of NICU admission. Male neonates have consistently shown higher rates of respiratory distress and perinatal complications [2, 3], which may explain their increased need for NICU admissions.

Instrumental delivery is the most common obstetric intervention, with vacuum‐assisted vaginal delivery (VAVD) and forceps being the most frequent. When used correctly, VAVD is safe, efficient, and it can help avoid cesarean sections [4, 5]. However, unsuccessful VAVD has been associated with neonatal complications such as cephalohematoma [6], subgaleal hemorrhage, spinal cord injury [5], and NICU admission [7]. Conversely, conditions such as prematurity, hypoglycemia, jaundice, respiratory distress, congenital malformations, and neonatal sepsis may necessitate NICU admission independently of VAVD [8] use.

Despite extensive international research, the safety of VAVD remains debated [9]. Several studies [6, 10–12] have reported no significant association between VAVD and adverse neonatal outcomes, whereas others [13–18] have demonstrated increased risks. This is due to discrepancies in the operator capability, the selection of cases, indications for use, and institutional protocols. Therefore, further research is required to clarify this relationship across several clinical settings.

This is particularly important in Lebanon because population‐specific data are lacking. There are no national guidelines regarding the use of VAVD in Lebanon; therefore, this technique remains underutilized causing the country to have one of the highest cesarean section rates in the Middle East and North Africa region [19]. Cultural perceptions [20], the increasing medicalized labor, and varying levels of trust between healthcare providers influence delivery decisions. Additionally, differences between public and private healthcare facilities further affect standards of obstetric care.

Although international literature has explored complications associated with VAVD [21–25], local data on NICU admissions are scarce. In the context of high cesarean section rates and the lack of standardized national recommendations, understanding the safety and outcomes of VAVD in Lebanon is crucial [26]. Accordingly, the objective of this study was to assess the association between VAVD and NICU admission among neonates delivered at a tertiary hospital in Lebanon, while taking relevant maternal, obstetric, and neonatal factors into account.

## 2. Methods

### 2.1. Study Design and Setting

The study followed a retrospective hospital‐based case‐control design and was conducted at the labor and delivery unit of Notre Dame des Secours University Hospital in Byblos, Lebanon between January 2021 and December 2023. This retrospective case‐control design allowed the identification of neonates with the outcome of interest (NICU admission) and comparison with those without the outcome to evaluate prior exposure to VAVD and other clinical factors recorded before admission.

### 2.2. Study Population

All women aged ≥ 18 years who delivered a singleton neonate during this study period were screened for eligibility. Prematurity is a well‐established factor influencing NICU admission; hence, we included term (≥ 37 weeks) and preterm (< 37 weeks) neonates in our study. Multiple gestations, stillbirths, outborn neonates, and major congenital anomalies incompatible with life were not included in the study. However, neonates with minor or surgically correctable congenital anomalies such as anal imperforation, choanal atresia, cystic hygroma, or spina bifida were included because they do not essentially threaten survival. This contributed to keeping a representative clinical presentation among those delivered via VAVD.

### 2.3. Case and Control Definitions

Cases included the neonates that were admitted to the NICU following delivery in this study period. Controls included neonates not admitted to the NICU. All the data and indications were collected from the neonatal medical records. A total of 488 deliveries were included in the study. The population was then divided according to the mode of delivery. A total of 200 neonates were born via VAVD, of whom 31 were admitted to the NICU and 169 were not; these VAVD subgroups were analyzed as part of the overall case (NICU) versus control (non‐NICU) framework.

### 2.4. Variables

We selected our variables after reviewing the existing literature and previous similar studies [10, 12–15, 18]. We examined maternal, obstetric, and neonatal characteristics. The mode of delivery was the primary exposure variable. We categorized it as VAVD or spontaneous vaginal delivery (SVD). Cesarean sections were analyzed separately as an independent variable and were excluded in the primary exposure comparison. Respiratory distress was coded as present when a clinical diagnosis of respiratory distress was recorded in the neonatal chart, whereas grunting was coded separately when explicitly documented as an isolated clinical sign.

### 2.5. Sample Size Calculation

Sample size was calculated using EPI INFO 7 StatCalc, assuming a case‐control design, a 95% confidence level, 80% power, and an expected odds ratio of 3.5. Data from a previous study suggested NICU admission rates of 35% among neonates born via VAVD compared with 12% among those who did not require NICU care. The minimal required sample size was 150 participants (30 cases and 120 controls). This represented the minimum number required to detect the hypothesized association between VAVD and NICU admission with 80% power and a 95% confidence level. However, during the study period, a total of 488 eligible deliveries were recorded in the hospital database, and all were included in the analysis. This increased the precision of estimates, improved the stability of multivariable models, and reduced the risk of bias that can arise from selectively excluding eligible participants.

### 2.6. Statistical Analysis

SPSS Version 27 was used to perform data analysis. Chi‐square tests were used to compare the categorical variables between neonates admitted to the NICU and those not admitted. This was used when expected cell counts were adequate, including gender, respiratory distress, maternal–fetal infection, prematurity, jaundice, mode of delivery, and other binary clinical characteristics. As for the categorical variables with small expected frequencies or rare events, such as spina bifida, anal imperforation, oligohydramnios, shoulder dystocia, and specific congenital anomalies, Fisher′s exact test was applied. Continuous variables, primarily gestational age (weeks at delivery) and other continuous maternal or neonatal measures, were analyzed using Student *t*‐test to compare mean values between the NICU and non‐NICU groups. Bonferroni correction was used to adjust for multiple testing in the bivariate analyses; the adjusted significance threshold was set at *p* ≤ 0.001, derived by dividing 0.05 by the 37 variables tested. Variables with Bonferroni‐adjusted *p* values ≤ 0.001 in bivariate analyses were entered into the multivariable logistic regression model. Statistical significance in the final model was defined as *p* < 0.05.

### 2.7. Ethical Considerations

Ethical approval was obtained from the Institutional Review Board of Centre Hospitalier Universitaire–Notre Dame des Secours (CR: 2/2024). As this was a retrospective review of anonymized records, the requirement for informed consent was waived. Because this was a retrospective review of anonymized routine clinical records, no additional interventions or contact with patients occurred as a result of including more than the minimum calculated sample size. The inclusion of all eligible deliveries during the study period did not introduce additional risk for participants and was considered ethically acceptable by the Institutional Review Board.

## 3. Results

### 3.1. Study Population Characteristics

During the study period, 488 deliveries were recorded, including 200 VAVDs (41%). Among neonates delivered via VAVD, 31 (18.6%) were admitted to the NICU and 169 (52.6%) were not. Overall, 167 of the total 488 neonates (34.2%) required NICU admission for various indications. A summary of the clinical characteristics of the total sample appears in Table 1.

**Table 1:** Characteristics of the study participants (*n* = 488).

Gestational age (weeks)	37.58 ± 1.79
Gender of the child	
Male	253 (51.8%)
Female	233 (47.7%)
Hypoglycemia (yes)	5 (1.0%)
Hypothermia (yes)	2 (0.4%)
Hypotonicity (yes)	1 (0.2%)
Bad sucking (yes)	4 (0.8%)
Hematemesis (yes)	1 (0.2%)
Anal imperforation (yes)	1 (0.2%)
Respiratory distress (yes)	57 (11.7%)
Maternal–fetal infection (yes)	46 (9.4%)
Premature (yes)	37 (7.6%)
Grunting (yes)	8 (1.6%)
Jaundice (yes)	16 (3.3%)
IUGR (yes)	2 (0.4%)
Apnea (yes)	1 (0.2%)
Anuria (yes)	1 (0.2%)
Oligohydramnios (yes)	3 (0.6%)
Ruptured axillary cystic hygroma (yes)	2 (0.4%)
Spina bifida (yes)	2 (0.4%)
Placental abruption (yes)	1 (0.2%)
Gestational diabetes (yes)	15 (3.1%)
Preeclampsia (yes)	21 (4.3%)
Vaginal bleeding (yes)	14 (2.9%)
PROM (yes)	32 (6.6%)
Oligohydramnios (yes)	3 (0.6%)
Shoulder dystocia (yes)	5 (1.0%)
Mode of delivery	
C‐section	127 (26.0%)
Vaginal delivery	361 (74.0%)
Forceps (yes)	9 (1.8%)
Vacuum (yes)	200 (41.0%)
NICU admission (yes)	167 (34.2%)

### 3.2. Bivariate Analysis

NICU‐admitted neonates had significantly lower mean gestational age compared with those not admitted. Preterm birth was significantly more common among NICU admissions. Neonates admitted to the NICU also had higher rates of respiratory distress, maternal–fetal infection, grunting, and jaundice. Mode of delivery differed significantly between groups: cesarean section deliveries were more frequently associated with NICU admission, whereas VAVD was more common in the non‐NICU group. Variables with statistically significant associations at the adjusted Bonferroni *p* value threshold (*p* ≤ 0.001) are highlighted in Table 2.

**Table 2:** Bivariate analysis of factors associated with NICU admission.

Variable	No NICU admission	NICU admission	*p*	Effect size
Gestational age	38.40 ± 0.99	36.01 ± 1.91	**< 0.001**	1.74
Gender of the child			0.251	0.053
Male	161 (50.2%)	92 (55.8%)		
Female	160 (49.8%)	73 (44.2%)		
Respiratory distress			**< 0.001**	0.504
No	321 (100%)	110 (65.9%)		
Yes	0 (0%)	57 (34.1%)		
Maternal–fetal infection			**< 0.001**	0.196
No	304 (94.7%)	138 (82.6%)		
Yes	17 (5.3%)	29 (17.4%)		
Premature			**< 0.001**	0.397
No	321 (100%)	130 (77.8%)		
Yes	0 (0%)	37 (22.2%)		
Grunting			**< 0.001**	0.179
No	321 (100%)	159 (95.2%)		
Yes	0 (0%)	8 (4.8%)		
Jaundice			**< 0.001**	0.255
No	321 (100%)	151 (90.4%)		
Yes	0 (0%)	16 (9.6%)		
Gestational diabetes			0.050	0.097
No	315 (98.1%)	158 (94.6%)		
Yes	6 (1.9%)	9 (5.4%)		
Preeclampsia			< 0.001	0.230
No	318 (99.1%)	149 (89.2%)		
Yes	3 (0.9%)	18 (10.8%)		
Vaginal bleeding			0.004	0.135
No	317 (98.8%)	157 (94.0%)		
Yes	4 (1.2%)	10 (6.0%)		
PROM			1	0.001
No	300 (93.5%)	156 (93.4%)		
Yes	21 (6.5%)	11 (6.6%)		
Delivery			**< 0.001**	0.783
C‐section	4 (1.2%)	123 (73.7%)		
Vaginal delivery	317 (98.8%)	44 (26.3%)		
Forceps			0.285	0.062
No	317 (98.8%)	162 (97.0%)		
Yes	4 (1.2%)	5 (3.0%)		
VAVD			**< 0.001**	0.329
No	152 (47.4%)	136 (81.4%)		
Yes	169 (52.6%)	31 (18.6%)		

*Note:* Means and standard deviations for continuous variables were compared between NICU‐admitted and nonadmitted neonates using Student *t*‐test, and proportions of categorical variables were compared using chi‐square or Fisher′s exact tests, as appropriate. Bold text indicates statistically significant *p* values after Bonferroni correction (*p* ≤ 0.001). Premature: delivery before 37 completed weeks of gestation. Mode of delivery (vaginal vs. C‐section) refers to spontaneous vaginal delivery compared with cesarean section. Vacuum‐assisted vaginal delivery (VAVD) is shown as a separate binary variable (yes/no). Respiratory distress: combined clinical diagnosis including signs such as tachypnea, retractions, and oxygen requirement. Grunting: presence of grunting documented as a specific physical sign, analyzed separately due to its distinct recording in medical files.

Abbreviations: aOR, adjusted odds ratio; CI, confidence interval; NICU, neonatal intensive care unit; PROM, premature rupture of membranes; VAVD, vacuum‐assisted vaginal delivery.

### 3.3. Multivariable Analysis

In the logistic regression model, higher gestational age was independently associated with lower odds of NICU admission (aOR = 0.39; 95% CI: 0.29–0.53; *p* < 0.001). Maternal–fetal infection significantly increased the likelihood of NICU admission (aOR = 5.59; 95% CI: 2.04–15.35; *p* < 0.001). Compared with cesarean section, vaginal delivery (SVD) was strongly associated with decreased NICU admission (aOR = 0.01; 95% CI: 0.002–0.24; *p* < 0.001). Finally, VAVD was not significantly associated with NICU admission (aOR = 2.11; 95% CI: 1.00–4.49; *p* = 0.051) (Table 3).

**Table 3:** Multivariable analysis: logistic regression taking the admission to NICU versus not∗ as the dependent variable (Nagelkerke *R* ^2^ = 0.760).

Variable	*p*	aOR	95% CI
Gestational age	**< 0.001**	0.39	0.29; 0.53
Maternal–fetal infection (yes vs. no∗)	**< 0.001**	5.59	2.04; 15.35
Mode of delivery (vaginal vs. C‐section∗)	**< 0.001**	0.01	0.002; 0.24
Vacuum‐assisted delivery (yes vs. no∗)	0.051	2.11	1.00; 4.49

*Note:* Asterisk “∗” denotes reference group. Bold text indicates statistically significant *p* values.

Abbreviations: aOR, adjusted odds ratio; CI, confidence interval.

## 4. Discussion

The study evaluated the association between VAVD and NICU admission in a tertiary hospital in Lebanon. The findings show that VAVD was not significantly associated with NICU admission, even after adjusting for the key maternal, obstetric, and neonatal variables. In contrast, gestational age, maternal–fetal infection, and mode of delivery emerged as primary predictors of NICU admission.

The absence of association between VAVD and NICU admission conforms to several international studies that suggest the safety and efficacy of VAVD if performed for the right clinical indications and by trained practitioners [27]. Many neonatal complications reported after VAVD are more closely related to the underlying indication for the intervention [28], such as fetal distress or maternal exhaustion, rather than the procedure itself. However, even though these factors are well‐recognized contributors to neonatal morbidity, they were not studied in detail because they were not the primary focus.

Gestational age was a strong predictor of NICU admission, reflecting the physiological vulnerabilities of premature neonates [29], including immature respiratory and thermoregulatory systems. The local hospital protocols recommend admitting neonates to the NICU if born before 35 weeks′ gestation. This further explains the significant association.

Maternal–fetal infection was also significantly associated with NICU admissions. This matches with a comprehensive literature showing that neonatal sepsis is one of the most frequent causes of NICU admission [30–32] in the world. Cesarean section deliveries were strongly associated with NICU admission compared with vaginal births. This is consistent with prior studies [33, 34] that have shown increased respiratory morbidity and adaptation difficulties among neonates born via cesarean section, particularly when performed before the onset of labor.

In our study, a notable proportion of cesarean sections are performed in emergency settings due to fetal distress, which further increases the risk of NICU admission. Moreover, even in the absence of clear complications, neonates born by cesarean section are mostly admitted to the NICU for observation because the Lebanese hospitals use a precautionary approach.

Overall, our results indicate that in our setting, gestational age, maternal–fetal infection, and particularly cesarean section as mode of delivery, are the main indications for NICU admission rather than VAVD itself. Optimizing the timing and indications of cesarean sections, early detection, and management of maternal–fetal infections, have greater impact on reducing NICU admissions rather than restricting the use of VAVD, which appears safe when performed appropriately.

### 4.1. Clinical Implications

This study discusses an important gap in the Lebanese obstetric literature. It provides local evidence regarding the safety of VAVD. The findings have several implications for clinical practice:

1. VAVD as a safe alternative:

Our study supports the appropriate use of VAVD through clear clinical criteria, operator training, and monitoring of outcomes. VAVD can be safely used as an alternative to cesarean section in properly selected cases because our study showed no association between VAVD and increased NICU admissions. This is important because the cesarean section rate in Lebanon is high, so increasing its appropriate use could help reduce the high cesarean section rates.

2. Guideline development:

Lebanon needs established standardized protocols to improve the safety and consistency in clinical decision‐making because there are no national guidelines for instrumental vaginal delivery. This likely contributes to the variations in practice.

3. Training and skill optimization:

Outcomes are strongly dependent on practitioner skill; hence, enhancing training in operative vaginal delivery may help optimize maternal and neonatal outcomes

4. Patient counseling:

These findings may help clinicians counsel expectant mothers, address misconceptions, and improve acceptance of VAVD as a safe intervention.

5. Health system impact:

In resource‐limited settings, the appropriate use of VAVD may reduce unnecessary NICU admissions and associated healthcare costs.

## 5. Limitations

There are several limitations in this study that should be considered when interpreting the findings. The study has a retrospective design and relies only on medical records, which may be incomplete or inaccurate, introducing risks of selection and information bias related to the documentation quality. Moreover, case‐control designs cannot establish causality between exposures and NICU admission. The minimal sample size calculated targeted 150 participants to achieve 80% power for the primary association of interest; however, the study included 488 eligible deliveries. We included all available neonates during the study period to increase the precision of odds ratio estimates, and allow a more robust adjustment for confounders, and a more representative picture of routine clinical practice. Additionally, the generalizability of the findings to all Lebanese regions was limited because the study was conducted in a single tertiary hospital. The population did not fully represent the diversity of obstetric practices across the country. Despite multivariable adjustments, residual confounding is also possible, as certain variables were not captured in the dataset (e.g., indication for VAVD or cesarean section, timing of intervention, operator experience, and socioeconomic factors) and therefore could not be included in the analysis. Since the indication for operative delivery may itself influence neonatal outcomes, residual confounding by indication cannot be excluded. Furthermore, some neonatal morbidities commonly associated with VAVD (cephalohematoma, subgalea hemorrhage, and spinal cord injuries) were not studied. This study cannot establish causality between VAVD and individual adverse outcomes because the primary outcome was NICU admission rather than neonatal complications. Future prospective studies should systematically include all obstetric variables and investigate the neonatal morbidities more comprehensively to assess the independent association between VAVD and NICU admission. Prospective multicenter studies are needed to validate these results, strengthen national evidence, and support the development of standardized guidelines for the safe and appropriate use of VAVD in Lebanon.

## 6. Conclusion

VAVD was not significantly associated with NICU admission in our hospital setting. The results are independent of gestational age, mode of delivery, and maternal–fetal infection. These findings provide a scientific basis for prioritizing prevention of infection, careful timing of cesarean sections, and wider, protocol‐guided use of VAVD. Scaling up trained and standardized VAVD practice within national guidelines could contribute to lowering cesarean section rates while maintaining safe neonatal outcomes.

## Author Contributions

M.A., S.H., and H.B. designed the study; M.A. drafted the manuscript; S.H. carried out the analysis and interpreted the results; M.A. and T.C. collected the data; H.B. and S.H. reviewed the paper for intellectual content. S.H. and H.B. contributed equally to this work and are last coauthors.

## Funding

No funding was received for this manuscript.

## Disclosure

All the authors reviewed the final manuscript and gave their consent.

## Ethics Statement

The Institutional Review Board in Centre Hospitalier Universitaire–Notre Dame des Secours, Jbeil, Lebanon, approved this research on July 12, 2024. (CR: 2/2024). The study′s methodology complied with all applicable rules and regulations (Declarations of Helsinki).

## Consent

The authors have nothing to report.

## Conflicts of Interest

The authors declare no conflicts of interest.

## Data Availability

The data that support the findings of this study are available on request from the corresponding author. The data are not publicly available due to privacy or ethical restrictions.
